# Rasputin/G3BP mediates subversion of antiviral immunity by o’nyong-nyong virus in *Anopheles coluzzii*

**DOI:** 10.1371/journal.ppat.1014423

**Published:** 2026-07-14

**Authors:** Solène Cottis, Adrien A. Blisnick, Emma Brito-Fravallo, Mariette Matondo, Kenneth D. Vernick, Anna-Bella Failloux, Christian Mitri

**Affiliations:** 1 Department of Parasites and Insect Vectors, Institut Pasteur, Université de Paris Cité, CNRS UMR2000, Genetics and Genomics of Insect Vectors Unit, Paris, France; 2 Graduate School of Life Sciences ED515, Sorbonne Universités UPMC Paris VI, Paris, France; 3 Department of Virology, Institut Pasteur, Université de Paris Cité, Arboviruses and Insect Vectors Unit, Paris, France; 4 Department of Structural Biology and Chemistry, Institut Pasteur, Université de Paris Cité, Mass Spectrometry for Biology Platform UTECHS, CNRS UAR2024, Paris, France; National Institutes of Health, UNITED STATES OF AMERICA

## Abstract

Cellular G3BP proteins are essential for alphavirus infection in both vertebrate and mosquito hosts, but the underlying mechanism of their proviral activity is poorly understood in any host. Whether the mosquito G3BP ortholog, Rasputin (*Rin*), interacts with host immunity to influence alphavirus infection has not been investigated, and anopheline mosquito interactions with arboviruses have been little studied. Here, we find that *Rin* silencing in *Anopheles* mosquitoes results in decreased ONNV infection levels, indicating a proviral activity for *Anopheles Rin*. We find that *Rin* function is required to maintain basal activity of the antiviral Imd and JAK/STAT pathways in uninfected mosquitoes. However, during ONNV infection, the control of the Imd pathway by *Rin* activity appears corrupted because *Rin* silencing leads to overexpression of the Imd positive regulator, *Rel2*. Thus, silencing of *Rin* both augments *Rel2* transcript abundance and decreases ONNV load. Co-silencing of *Rel2* with *Rin* restores normal ONNV infection levels, indicating that *Rin* activity is required to inhibit Imd function during ONNV infection, and which explains most of the *Rin* proviral phenotype. In addition, we show that the ONNV non-structural protein 3 (nsP3), which binds to Rin, strongly alters the pattern of *Anopheles* cellular protein partners interacting with Rin. In the presence of ONNV nsP3, 48 Rin-binding host proteins are unchanged but seven binding proteins are excluded and eight new cellular proteins bind Rin. The altered cellular protein partners are candidate host factors involved in viral subversion of Rin control over Imd activity. Overall, these results reveal a molecular mechanism in which ONNV, probably through nsP3, co-opts the normal Rin function for basal cellular immune activity by subverting the Imd antiviral pathway to promote infection. These results may be generalizable for Rin function during alphavirus infection of other mosquitoes, as well as for G3BP function in the mammalian host, and could offer a target for development of vector-based genetic control tools against arbovirus transmission.

## Author summary

Understanding antiviral mechanisms in potential new arbovirus vectors, such as *Anopheles* mosquitoes, is essential for assessing risks related to arbovirus spread by vector shift. Using an arbovirus naturally transmitted by *Anopheles*, we discover a proviral role for the mosquito G3BP, Rin, whose function in controlling basal immune responses is subverted by ONNV infection. This finding, to our knowledge, is the first concrete evidence of Rin interaction with immune regulation and its hijacking by an arbovirus in mosquitoes. We also identified *Anopheles* Rin protein partners, which pattern is altered by the non-structural protein 3 of the ONNV. These findings pave the way for new mosquito antiviral protein candidates, which could be targeted to curb arbovirus transmission.

## Introduction

Arthropod-borne viruses (arboviruses) are maintained through an alternating cycle of transmission between the vertebrate and arthropod vector hosts. Arboviruses represent a spreading global burden for human and animal health, with the clinically most important pathogens represented by RNA viruses in the families *Flaviviridae*, *Togaviridae*, *Bunyavirales*, and *Reoviridae* [1]. The alphavirus o’nyong-nyong virus (ONNV, genus alphavirus, family *Togaviridae*) is closely related to chikungunya virus (CHIKV, genus alphavirus, family *Togaviridae*). Both are in the same antigenic group, the Semliki forest virus complex, and cross-reactivity between antibodies against related viruses causing similar symptoms in humans makes serological tests difficult to interpret [2–4]. The most apparent difference between the arboviruses is their transmission to humans by distinct mosquito vectors (reviewed in [5]). *Anopheles* mosquitoes are the major vector of human malaria but are the primary vector of only one known arbovirus, ONNV. Other arboviruses, such as Rift Valley fever virus [6] have occasionally been isolated in nature from *Anopheles* (reviewed in [7]), although the significance of these observations for transmission is not known. Nevertheless, *Aedes* mosquitoes are known to be the primary vectors of many arboviruses, including CHIKV.

The conserved host proteins in the family of Ras-GTPase-activating protein (SH3 domain)-binding proteins (G3BPs), and the mosquito ortholog Rasputin (*Rin*) [8], are proviral factors for alphavirus infection. Rin/G3BPs are enigmatic molecules with multiple biological functions [9,10]. Most studies of Rin/G3BP function have been carried out in mammalian cells, where three isoforms (G3BP1, G3BP2a and 2b) are expressed from two genes [10,11]. Mosquitoes carry a single *Rin* gene, and the few published Rin studies to date have only been in *Aedes* mosquitoes [12–14], whereas, to our knowledge, there are no previous reports about Rin functional activity in *Anopheles* mosquitoes.

Mammalian G3BPs are involved in numerous biological processes including RNA-binding, RNA metabolism, stress granule formation, and regulation of ubiquitin-mediated degradation signaling [9–11,15–17]. Rin/G3BPs can interact and colocalize with the non-structural protein 3 (nsP3) of many alphaviruses in *Aedes* and mammalian cells, and the interaction with nsP3 may sequester Rin/G3BPs away from their usual cytoplasmic location into cytoplasmic stress granules [12–14,17,18]. Rin/G3BPs also serve as proviral host factors promoting the infection cycle of multiple RNA viruses in mammals, while in *Aedes*, proviral activity has been shown so far only for alphaviruses [12]. The mechanism of proviral function of Rin/G3BPs is not clearly understood [19]. Interestingly, G3BPs were reported to potentially interact with vertebrate host immunity by influencing cytoplasmic distribution of the regulators of the NF-kappa B pathway [20], by enhancing NF-kappa B phosphorylation and protein quantity [19,21], and by modulating NF-kappa B signaling through protein kinase R or by other means [22,23], although the potential interaction of Rin/G3BPs with host immunity has not been examined in detail in either the mammalian or insect host. To our knowledge, there are no reports in mosquitoes of potential Rin/G3BP interaction with immune regulation.

The *Anopheles* antiviral response against ONNV was previously shown to be physiologically compartmentalized, based on distinct protective pathways during the different phases of the infection [24]. Host immunity during the primary infection of the midgut epithelium is dominated by the Imd and JAK/STAT pathways, while in contrast, during the systemic disseminated infection after midgut escape, beginning about 3 d post-bloodmeal, the RNAi and Toll pathways dominated the antiviral defense. In the current study, we identified a functional link between Rin activity and antiviral immunity after ONNV bloodmeal infection. We found that Rin activity is proviral for ONNV infection, and we hypothesized that the proviral activity could be explained if Rin is required for host immune signaling integrity, and if a viral virulence factor can manipulate Rin to undermine cellular immune signaling in relevant antiviral pathways. We found that in uninfected mosquitoes, Rin is required for basal immune activity. In mosquitoes challenged with ONNV, Rin activity appeared corrupted, which leads to inhibition of the Imd pathway. Co-silencing of *Rin* and *Rel2* complements the loss of Rin and restores ONNV infection levels, consistent with a model where Rin is manipulated by ONNV to inhibit Imd, and where depletion of Imd removes the viral need for Rin as a proviral factor to achieve normal infection levels. The ONNV protein nsP3 physically binds Rin in *Anopheles* cells, and modifies the pattern of cellular protein partners interacting with Rin. Thus, Rin influences *Anopheles* host defense against ONNV infection and is probably a target for viral manipulation by nsP3 in order to subvert antiviral immunity. Beyond *Anopheles* and ONNV, these results are likely relevant to the general understanding of G3BPs in mammals and other alphaviruses.

## Results

### *Anopheles* Rin activity is proviral for ONNV and influences multiple immune pathways in mosquitoes

**Fig 1:**
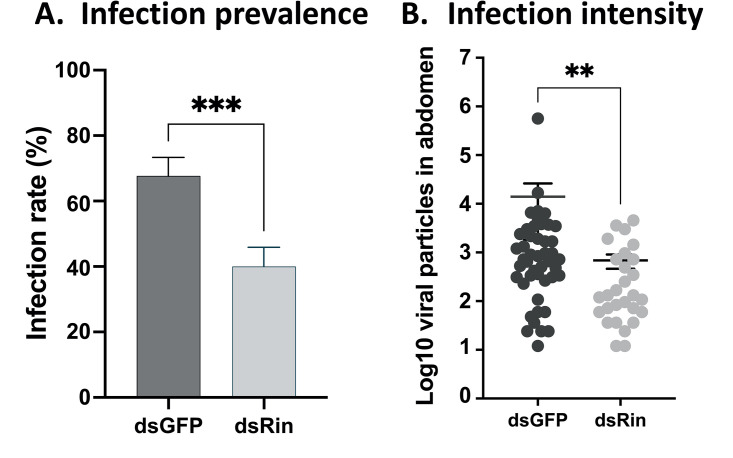
Rin is a proviral factor for ONNV infection in *Anopheles coluzzii* mosquitoes. Female mosquitoes were treated with dsRin or dsGFP two days prior to an infectious bloodmeal. ONNV infection was measured 3 d post-bloodmeal by viral titration. **(A)** The effect of *Rin* silencing on infection prevalence among bloodfed mosquitoes as a proportion of the total mosquitoes analyzed. y-axis, percentage of mosquitoes positive for ONNV in dsRin or dsGFP-treated mosquitoes. **(B)** The effect of *Rin* silencing on infection intensity in the positive mosquito samples. y-axis, load of viral particles, each point represents the viral titer in one mosquito. Error bars indicate the SEM. Results are from three independent replicates, n = 72 per condition. Analysis of infection prevalence was performed using a chi-squared analysis test. A two-tailed non-parametric unpaired Mann-Whitney test was performed to assess the statistical significance of the difference in viral titer in mosquitoes. The P-values were considered significant if p < 0.05 (** P < 0.01, *** P < 0.005, ns non-significant).

Rin/G3BP is proviral for infection of many alphaviruses in mammalian cells and for infection of CHIKV in *Aedes albopictus* [12,19,25] but the role of *Anopheles* Rin on viral infection has not been examined. Therefore, we first tested whether Rin is proviral for ONNV in *Anopheles coluzzii* mosquitoes by silencing *Rin* transcript using RNAi-mediated gene silencing. Double-stranded RNA (dsRNA) specific for *Rin* (dsRin), or irrelevant GFP control (dsGFP) were injected into mosquitoes followed by bloodmeal infection with ONNV. Unfed females were removed, and ONNV infection in abdomens was measured by viral titration 3 days (d) post-bloodmeal. We found that *Rin* silencing significantly decreased ONNV infection prevalence (defined as the proportion of fully fed mosquitoes positive for ONNV) (Fig 1A) and infection intensity or titer (defined as the viral load measured only in the ONNV-positive mosquitoes) (Fig 1B). This result indicates that Rin displays a proviral activity for ONNV in *An. coluzzii*.

We next determined whether Rin influences basal immunity in uninfected mosquitoes by measuring the expression of specific components of Imd, Jak/STAT, RNAi, and Toll pathways.

**Fig 2:**
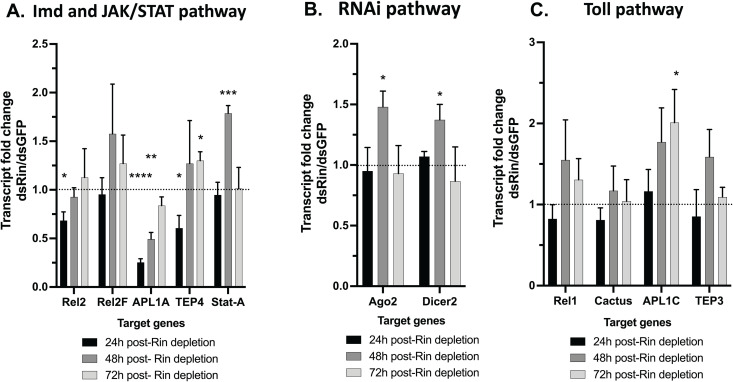
Rin function is required for transcriptional basal immunity in uninfected *Anopheles* mosquitoes. Female mosquitoes were treated with dsRin or control dsGFP. The influence of *Rin* silencing at 24, 48 and 72h post-injection on gene transcript abundance is shown for (**A**) the Imd and JAK/STAT pathways, (**B**) the siRNA pathway, and (**C**) the Toll pathway. For all panels, x-axis indicates dsRNA treatments. Each bar represents the level of transcript abundance of each immune gene relative to dsGFP-treated controls (defined as 1.0), and error bars indicate the SEM. Results are from three independent replicates, n = 18 per condition. Student t-test analysis, which compares the mean of two independent groups (control and treatment) were used to assess the statistical significance of the difference in transcript abundance. The P-values were considered significant if p < 0.05 (*P < 0.05, ** P < 0.01, *** P < 0.001, **** P < 0.0001).

Silencing of *Rin* in naïve mosquitoes displayed a striking effect upon the Imd and JAK/STAT pathways (Fig 2A), which were both shown to be efficient antiviral pathways against ONNV in *Anopheles* [24]. In the Imd pathway, *Rin* silencing caused decreased transcript abundance of the Imd positive regulator transcription factor *Rel2* and two Imd pathway effector genes, the leucine-rich repeat protein *APL1A* and the thioester-containing protein *TEP4*. Both *APL1A* and *TEP4* are transcriptionally regulated by Rel2 but not by the Toll pathway positive regulator Rel1 [26–28]. Therefore, APL1A and TEP4 are informative specific reporters for Imd pathway activity, and at least APL1A is antiviral for ONNV in Anopheles (TEP4 was not tested) [24]. *Rel2*, *APL1A* and *TEP4* displayed a gradual tendency of increased transcript levels from 24 h to 72 h post-*Rin* silencing, but which was not significant for any except for *TEP4* at 72 h, for an unknown reason. In the JAK/STAT pathway, *Rin* silencing caused increased transcript abundance of the JAK/STAT positive regulator, *Stat-A*, and therefore Rin is required to limit *Stat-A* transcript to basal levels. Thus, silencing of *Rin* causes widespread dysregulation of the Imd and JAK/STAT pathways, and normal Rin function is required for controlling basal activity of these immune pathways.

The siRNA pathway has been studied for its role in counteracting mosquito infection by numerous RNA viruses. The endonucleases Argonaute-2 (Ago2) and Dicer-2 (Dcr2) are essential catalytic components of the RNA-induced silencing complex [29], and Ago2 was previously shown to be antiviral for ONNV infection in *Anopheles* [24]. The transcript abundance of *Ago2* and *Dicer2* both increased 48h after *Rin* silencing, and therefore Rin acts to limit the expression of the siRNA pathway in uninfected mosquitoes (Fig 2B). Finally, for the Toll pathway, we tested the expression of the transcription factor *Rel1*, the Toll negative regulator *Cactus* as well as effector genes regulated, at least in part, by the Toll pathway, *APL1C* and *TEP3* [26] (Fig 2A). Silencing of *Rin* does not cause a significant effect on Toll pathway-related transcript abundance, except for causing a late increase of *APL1C* transcript (Fig 2C).

Taken together, these results from uninfected *An. coluzzii* indicate that, among these four major arms of mosquito innate immunity, Rin significantly influences the basal activity of key components mainly in the Imd and JAK/STAT pathways. Thus, Rin is required to maintain the overall integrity and homeostasis of the basal immune readiness of mosquitoes in the unstimulated resting state, including of known antiviral factors for ONNV in *Anopheles*, consistent with the hypothesis that Rin proviral function could be linked to viral manipulation of host immunity.

### *Anopheles* Rin mediates inhibition of the Imd pathway during ONNV infection

We next examined the influence of Rin on *Anopheles* immunity to ONNV after challenge with an ONNV infective bloodmeal. Transcript abundance of the immune gene panel was measured at 2 d and 3 d after the infective bloodmeal.

**Fig 3:**
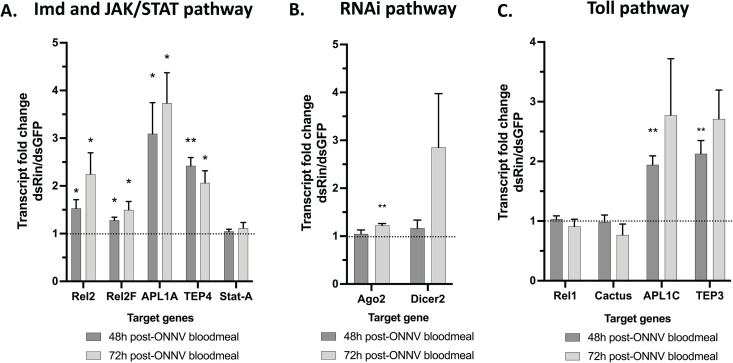
Rin is required for ONNV-dependent inhibition of the *Anopheles* Imd pathway. Female mosquitoes were treated with dsRin or control dsGFP and two days later were fed an ONNV infectious blood meal. The influence of *Rin* silencing is shown for (**A**) the Imd and JAK/STAT pathways, (**B**) the siRNA pathway, and (**C**) the Toll pathway. For all panels, x-axis indicates dsRNA treatments. Each bar represents the level of transcript abundance of each immune gene relative to dsGFP-treated control (defined as 1.0), and error bars indicate the SEM. Results are from three independent replicates, n = 48 per condition. Student t-test analysis, which compares the mean of two independent groups (control and treatment) were used to assess the statistical significance of the difference in transcript abundance. The P-values were considered significant if *P < 0.05, ** P < 0.01, *** P < 0.001, ns non-significant.

*Rin* silencing during the primary midgut infection of ONNV increases the transcript levels of genes in three of the four major immune signaling pathways, as compared to control mosquitoes treated with dsGFP (Fig 3A-3C). The major influence was observed in the Imd pathway, where the Imd positive regulators *Rel2* and *Rel2F*, and the anti-ONNV factor *APL1A* [24] are significantly induced by *Rin* silencing (Fig 3A). Thus, unlike in uninfected mosquitoes, during ONNV infection Rin function acts to decrease transcript levels of these antiviral genes. Interestingly, although the transcript level of the JAK/STAT positive regulator, *Stat-A*, is regulated by Rin in uninfected mosquitoes (Fig 2A), ONNV infection alters *Stat-A* regulation by abolishing Rin control over *Stat-A*. In addition, similarly to naïve mosquitoes, expression of the major regulator of the RNAi pathway, *Ago2*, remains significantly induced by *Rin* silencing during infection, although the effect is more significant during infection (Fig 3B). Finally, neither the positive (*Rel1*) nor negative (*Cactus*) regulators of the Toll pathway were influenced by absence of Rin activity during infection (Fig 3C). Two immune effectors thought to be regulated by Toll, *APL1C* and *TEP3*, were influenced by silencing of *Rin* during infection. The reason for this is not clear given the absence of a Rin effect on the Toll pathway regulators, and may indicate a previously unrecognized co-regulation of these effectors by pathways other than Toll.

Overall, and unlike in uninfected mosquitoes, the most important effect of *Rin* silencing during ONNV infection appeared to be *Rel2* overexpression, indicating that Rin activity inhibits the induction of *Rel2* only in the presence of virus infection. This suggests that Rin could be serving as a target for viral manipulation, in order to inhibit the potent antiviral Imd pathway.

### Rin proviral activity is mediated by its inhibition of Imd function during ONNV infection

**Fig 4:**
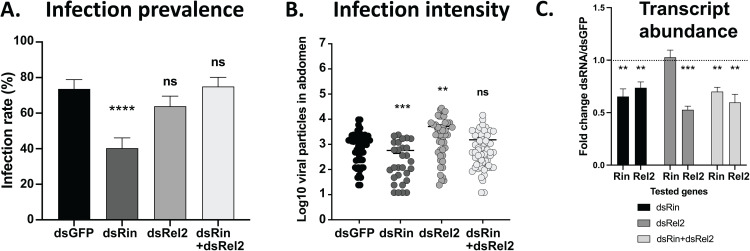
Rin proviral activity is abolished by co-silencing of the Imd pathway during ONNV infection. Mosquitoes were treated with the indicated dsRNAs 2 d prior to an ONNV infectious bloodmeal. Fully engorged mosquitoes were sampled at 3 d post-bloodmeal to measure ONNV infection by viral titration. **(A)** ONNV infection prevalence, the proportion of blood-fed mosquitoes positive for infection was measured. Bars indicate the percentage of mosquitoes positive for ONNV. **(B)** ONNV infection intensity was measured as the viral titer of only positive mosquitoes (uninfected mosquitoes were removed from the determination of intensity). Points represent the viral titer in one mosquito. **(C)** Gene silencing efficiency was confirmed on the day of infectious blood feeding for *Rin* and *Rel2* transcript abundance (S1 File). Bars represent the level of transcript abundance of *Rin* and *Rel2* relative to the dsGFP-treated control (defined as 1.0). For all panels, x-axis indicates dsRNA treatments. Results were from three independent replicates (A, B) with n = 72 mosquitoes per condition or (C) a pool of at least 6 mosquitoes per replicate. Analysis of infection prevalence was performed using a chi-squared analysis test. A two-tailed non-parametric unpaired Mann-Whitney test was performed to assess the statistical significance of the difference in viral titer in mosquitoes. Student t-test analysis, which compares the mean of two independent groups (control and treatment) were used to assess the statistical significance of the difference in transcript abundance. The P-values were considered significant if p < 0.05 ** P < 0.01, *** P < 0.001, **** P < 0.0001, ns non-significant. Error bars indicate SEM.

We showed above that Rin activity during ONNV infection displays an inhibitory effect upon the Imd pathway, which has potent antiviral activity against ONNV [24]. Therefore, we tested whether the control by Rin of Imd pathway activation could explain Rin proviral activity. Silencing of either *Rin* or *Rel2* alone, or co-silencing of *Rin* and *Rel2*, were carried out in mosquitoes followed by bloodmeal challenge with ONNV. Virus levels were measured in individual mosquitoes at 3 d post-bloodmeal by viral titration of abdomens and detection of focus-forming units (FFU). Silencing of *Rin* significantly decreased the proportion of ONNV-infected mosquitoes (infection prevalence; Fig 4A) as well as the number of ONNV particles (infection intensity or titer; Fig 4B) as compared to the dsGFP-treated control group. Uninfected mosquitoes were removed from the measurement of infection intensity to avoid conflating prevalence and intensity measures. Silencing of *Rel2* alone did not alter infection prevalence (Fig 4A), but did cause a significant increase in mosquito viral titers (Fig 4B), consistent with our previous observation for *Rel2* and ONNV infection [24]. Thus, ONNV infectivity to *Anopheles* is compromised by silencing of *Rin* activity, indicating that Rin activity is required for full ONNV infection levels, and is enhanced by silencing of *Rel2*.

To query whether the proviral activity of Rin could potentially act by subverting Imd antiviral protection during ONNV infection, *Rin* and *Rel2* were simultaneously silenced, followed by an ONNV infectious bloodmeal. The loss of Rel2 complemented *Rin* silencing and restored normal levels of infection prevalence as well as viral load, as compared to *Rin* silencing alone (Fig 4A & 4B). Thus, with *Rel2* silenced, Rin no longer displays detectable proviral activity for ONNV, because infection prevalence and intensity of the double silencing are the same wild-type level as the dsGFP controls with normal Rin and Rel2 activity. This outcome indicates that achieving a normal level of ONNV infection is independent of Rin activity when *Rel2* is silenced, and suggests that the presence of Rin is required for virus-dependent suppression of Imd pathway activity. Such a mechanism is further supported by examining the directionality of transcriptional control between *Rin* and *Rel2* (Fig 4C). Silencing of *Rin* significantly decreases its own transcript abundance and also that of *Rel2*, while silencing of *Rel2* decreases its own transcript but has no effect on *Rin* transcript abundance. Thus, Rin controls Rel2 by a hierarchical and unidirectional mechanism of transcript regulation, in which Rel2 does not branch back to influence *Rin* transcript levels.

Further detail is provided by examination of the infection phenotype caused by silencing of *Rel2* alone (Fig 4A). Rel2 activity limits viral titer, but alone does not appear to reduce infection prevalence, although the high (80%) infection prevalence in the dsGFP controls probably does not provide statistical power to detect a significant effect in the dsRel2 treatment alone, in contrast to the phenotype produced by silencing of *Rin* alone. This suggests that the nearly double rate of mosquitoes displaying sterile immunity for ONNV (i.e., absence of detectable infection) after *Rin* silencing may require the combined contribution of additional antiviral mechanisms that are also controlled by Rin, and cannot be explained only by Rin control over Rel2 alone.

These results confirm a proviral role for Rin during the infection of *An. coluzzii* with ONNV after an infectious bloodmeal. However, the Rin proviral phenotype requires an intact Imd pathway. In contrast, suppression of the Imd pathway by silencing of positive regulator *Rel2* rescues full ONNV infection even in the absence of Rin, indicating that Rin is unnecessary as a proviral factor in mosquitoes without an active Imd pathway. The simplest interpretation for these observations is that viral co-optation of normal Rin cellular function, particularly including its role in homeostasis of basal immunity, can subvert at least the important Imd, and potentially other antiviral immune pathways.

### Rin biochemically interacts with ONNV and its non-structural protein nsP3 in *Anopheles* cells

**Fig 5:**
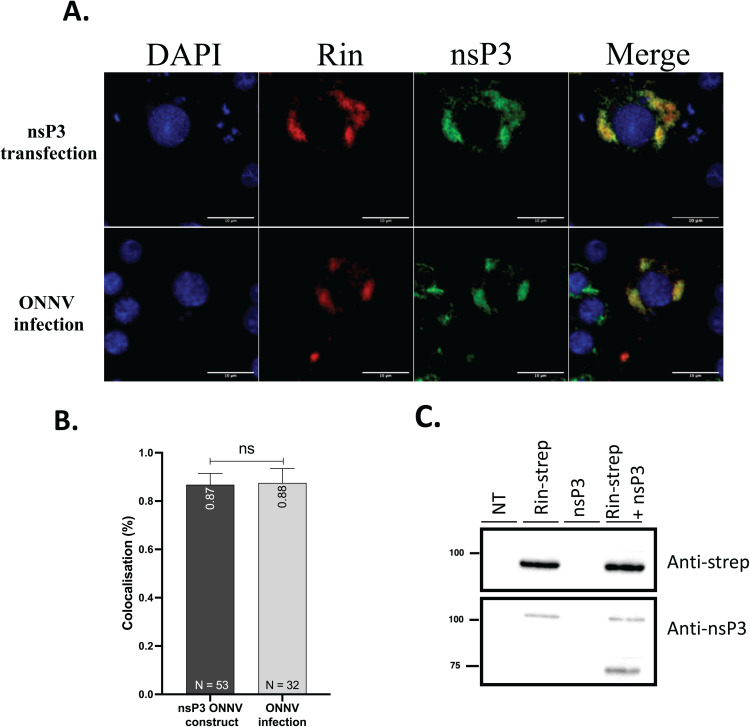
Rin biochemically interacts with ONNV and nsP3 in *Anopheles* cells. Immunostaining analysis of Rin colocalization with nsP3 and ONNV in *Anopheles* cells. **(A)** Top panel (nsp3 transfection): *Anopheles* cells were co-transfected with a C-terminal streptavidin-tagged construct of *Anopheles* Rin (Rin-strep) and a construct expressing nsP3 of ONNV, both driven by the *Anopheles* actin promoter. Rin was detected using streptavidin Strep-Tactin conjugated with dye-649 (red, #2-1568-050, IBA) and nsP3 was detected using a polyclonal antibody against ONNV nsP3 plus Alexa Fluor 555 conjugated secondary antibody (green) (#A27039, Invitrogen). Lower panel (ONNV infection): *Anopheles* cells were co-transfected with a C-terminal streptavidin-tagged construct of *Anopheles* Rin (Rin-strep), but not the construct expressing nsP3 of ONNV, and were infected with ONNV as the source of nsP3. Rin was detected using streptavidin Strep-Tactin conjugated with dye-649 (red), and the viral-source nsP3 was detected using the same polyclonal antibody against ONNV nsP3 plus Alexa Fluor 555 conjugated secondary antibody (green). Both panels, nuclei were stained with Hoechst 33342 dye (blue). Scale bars, 10 µm. Representative images are shown. **(B)** The colocalization of Rin and ONNV nsP3 signal was compared for transfection-source nsp3, or viral-source nsP3, by counting cells stained as above. 87% or 88% of cells counted, respectively, displayed colocalization of Rin and ONNV nsP3 signal under each condition of nsP3 source. There was no significant difference in colocalization for the two conditions of nsP3 source. ns, non-significant. **(C)** Streptavidin pull-down was used to affinity purify Rin-strep (Rin-strep) from 4a3A cells co-transfected with untagged nsP3, used as bait for nsP3 cellular partners. The eluates of the pull-down were analyzed by Western blot. Rin were detected using a streptavidin mouse monoclonal antibody conjugated with HRP, and nsP3 was detected using the same polyclonal antibody as above against ONNV nsP3 plus HRP-conjugated anti-rabbit secondary antibody. The top panel shows positive control detection of the expected Rin-strep band of 89 kDa detected by anti-streptavidin only in the cells transfected with the Rin-strep plasmid (lanes 2 and 4). The bottom panel shows the presence of a 70 kDa band corresponding to ONNV nsP3, which was only seen in the elution from cells co-transfected with Rin-strep and nsP3 (lane 4), but was not observed in the non-transfected cells (NT, lane 1), nor in cells transfected only with Rin-strep but not nsP3 (lane 2), nor in cells transfected only with nsP3 but not Rin-strep (lane 3). An unknown band of 110 kDa was also stained by the nsP3-polyclonal antibodies. NT, non-transfected control cells, Rin-strep, cells transfected only with Rin-strep, nsP3, cells transfected only with nsP3, Rin-strep + nsP3, cells co-transfected with both plasmids.

In mammalian and *Aedes* mosquito cells, G3BPs and Rin have been shown to colocalize with the non-structural protein 3 (nsP3) of multiple alphaviruses including CHIKV and ONNV [14]. However, to our knowledge *Anopheles* Rin has not been examined. Here, we analyzed the colocalization and interaction of ONNV nsP3 with *An. coluzzii* Rin in 4a3A cells (Fig 5). To measure colocalization, we co-transfected a C-terminal streptavidin tagged construct of *Anopheles* Rin (Rin-strep) and a construct expressing nsP3 of ONNV, with expression of both driven by the *Anopheles* actin promoter (Fig 5A, top panel, nsP3 transfection). Colocalization of Rin and virally-produced nsP3 was also tested after direct infection of cells with ONNV, without nsP3 transfection (Fig 5A, lower panel, ONNV infection). At least 87% of cells counted displayed colocalization of Rin and ONNV nsP3 signal with both nsP3 sources (Fig 5B). There was no significant difference in colocalization between Rin and transfected nsP3 in uninfected cells, and viral origin nsP3 in ONNV-infected cells.

To confirm the interaction between Rin and nsP3, we used a streptavidin-pull down assay to purify Rin conjugated with streptavidin (Rin-strep) from *Anopheles* 4a3A cells co-transfected with untagged nsP3. The resulting Western blots were detected with antibodies directed against nsP3, or as a positive control against streptavidin (Fig 5C). A 70 kDa band corresponding to ONNV nsP3 was only seen in the elution from Rin-strep in co-transfected cells but was not observed in the eluate of only nsP3- or Rin-strep transfected cells, indicating that nsP3 was bound and pulled down by Rin-strep. An unknown non-specific band of 110 kDa was also stained by the nsP3-polyclonal antibodies. The expected control band of 89 kDa was detected by anti-streptavidin in the cells transfected with Rin-strep construct. Therefore, in *Anopheles* 4a3A cells, Rin forms a stable physical interaction with ONNV nsP3, either by direct binding or indirectly through accessory binding proteins.

### ONNV nsP3 interaction with Rin alters the Rin-associated protein complex

ONNV infection alters Rin activity and causes Rin-dependent inhibition of Imd. ONNV nsP3 interacts stably with Rin in *Anopheles* cells, as it does with Rin/G3BPs in general, and this interaction is likely to be involved in the coopting of *Anopheles* Rin function. To shed light on the mechanism by which Rin regulates host immunity, we analyzed by mass spectrometry the Rin and Rin/nsP3 binding complex in *Anopheles* 4a3A cells. We first confirmed proviral activity of Rin in 4a3A cells, as was seen in mosquitoes, and found that silencing of Rin decreased the abundance of intracellular ONNV genomic RNA (S1A Fig) and infectious particles released into the supernatant (S1B Fig). *Rin* silencing in 4a3A cells was efficient (S2 Fig).

**Fig 6:**
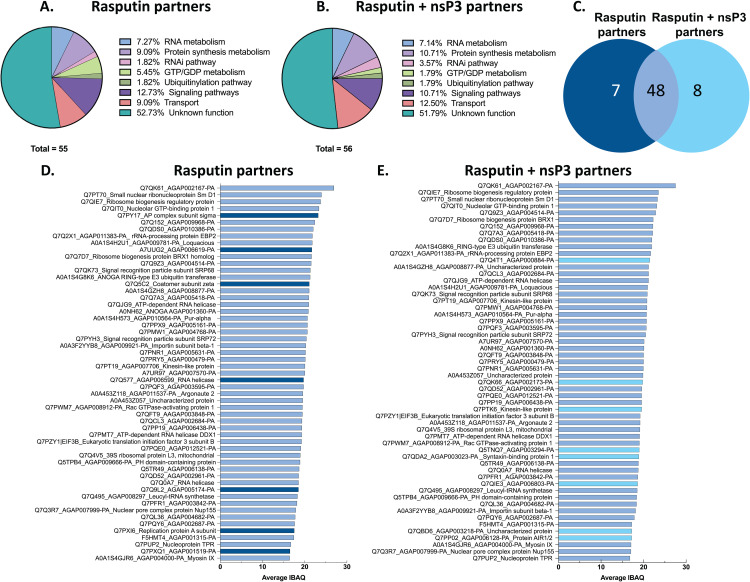
ONNV nsP3 binding alters Rin protein partners in *Anopheles* cells. Eluates of cells transfected with Rin-strep or co-transfected with Rin-strep and nsP3 of ONNV (as in Fig 5C) were analyzed by mass spectrometry to identify protein partners. The proteins identified in the control conditions were classified as non-specific and were removed from the analysis. **(A)** Summary of the biological functions of the protein partners of Rin-strep, or (**B**) of Rin-strep in presence of nsP3 are shown. **(C)** Graph indicates the partner proteins in common between Rin-strep and the complex Rin-strep/nsP3. **(D-E)** The average abundance (Intensity-Based Absolute Quantification, IBAQ) scores of the protein partners of Rin-strep (dark blue) or Rin-strep/nsP3 complex (light blue) or their protein partners in common (plain blue) are indicated. Each bar represents the average IBAQ abundance of each protein partner of Rin-strep or of the complex Rin-strep/nsP3. Data are from four replicates per condition. Raw data and statistical analyses of mass spectrometry analysis are presented in S2 File.

We then captured Rin and Rin/nsP3 binding complexes, using as bait Rin tagged with C-terminal streptavidin, transfected alone (Rin-strep) or co-transfected with ONNV nsP3 (Rin-strep+nsP3) (Fig 6, raw blot data in S3 Fig). Mass spectrometry was also carried out upon extensive controls for binding specificity to exclude irrelevant interactions, as follows: i) pulldown from non-transfected cell extract, to control for nonspecific protein binding to the Strep-Tactin magnetic beads, ii) pulldown from extract of cells transfected with streptavidin conjugated to the irrelevant protein, GFP (GFP-strep), to control for cellular proteins binding either to streptavidin, to the beads, and/or displaying general nonspecific protein binding activity, iii) pulldown from extract of cells transfected only with nsP3 without Rin-strep, to control for any binding of nsP3 without the streptavidin moiety to the beads, thus pulling down irrelevant proteins. Proteins found in the control conditions were considered non-specific and were not analyzed further.

Most Rin partners discovered in the proteomic screen are proteins with unknown function. Of the known proteins, Rin interacts in pathways related to RNA metabolism, protein synthesis components, ubiquitination pathways and signaling (Fig 6A; Raw data and statistical analyses of mass spectrometry analysis are presented in S2 File). The biological pathways of Rin partners are consistent with reported Rin involvement in Ras metabolism [30] and RNA metabolism [17]. However, when Rin is in complex with viral nsP3, the profile of protein partners is altered (Fig 6B). A total of 48 proteins interact with Rin in the presence or absence of nsP3, seven of them interacting only with Rin alone and 8 proteins interacting with Rin only when nsP3 is present (Fig 6C). These 8 new proteins become strong candidates to explain the biological effect of ONNV upon Rin function. The Rin-nsP3 complex includes 8 new protein partners that may be involved in protein synthesis, RNAi pathway and cellular/nuclear transport, although most of these proteins (5/8) have unknown function, and will require further work to understand, and fewer partners involved in GDP/GTP metabolism and in signaling pathways (Fig 6D and 6E). Therefore, the presence of nsP3 modifies the biological profile of Rin-interacting protein partners, likely in order to modify the function of Rin during ONNV infection.

## Discussion

Here we show that the mosquito G3BP, called Rin, influences important pathways of immunity including antiviral defenses in *Anopheles* mosquitoes. Rin activity is necessary for normal basal activity of the strongly antiviral Imd and JAK/STAT pathways in uninfected mosquitoes. In ONNV-infected mosquitoes, Rin is required for virus-dependent inhibition of Imd function during ONNV infection. This effect of Rin during infection explains most of the observed Rin proviral phenotype, and is most simply explained as a mechanism of viral subversion of host antiviral immunity through manipulation of Rin. Our results point to viral nsP3 alteration of the composition of the Rin functional protein complex as a mechanism underlying at least part of the observed viral suppression of host immunity.

In non-infected mosquitoes Rin activity upregulates the transcript levels of mainly Imd immune genes as compared to mosquitoes with *Rin* silenced, in which transcripts of the same immune genes are significantly less abundant. In contrast, during ONNV infection, Rin activity results in decreased transcript levels for these immune genes, as compared to *Rin*-silenced mosquitoes. Therefore, the normal Rin physiological function in naïve mosquitoes is corrupted by ONNV infection. Rin is proviral for ONNV infection in *Anopheles*, and the proviral phenotype is reversed by silencing of the Imd NF-kb positive regulator, Rel2. This result indicates that the Rin proviral function is correlated with and probably explained by its ability to regulate the Imd pathway. Moreover, there is a hierarchical relation between Rin and Rel2 such that Rin is able to regulate the transcript level of Rel2, but Rel2 does not branch back to regulate Rin transcript abundance. This observation indicates a directional polarity in which Rel2 is functionally downstream of Rin.

Rin displays an effect on both infection prevalence, the fraction of mosquitoes carrying any detectable infection, and on infection intensity, the viral titer in infected mosquitoes. The simplest model to explain the relationship between infection prevalence and viral titer could be that the activity of predominantly the Imd pathway reduces the efficiency of viral replication in the primary midgut infection below a level that can consistently yield an established infection, and sterile immunity then results in those individuals where a combination of low viral titer and the action of other antiviral and stochastic effects allows elimination and curing of the viral infection. The individuals able to cure the infection, resulting in sterile immunity, could either be random, where sterile immunity results from immunity and stochastic effects, or there could be a genetic contribution from the mosquito host, such that genetic polymorphism of certain variants in the mosquito colony may reduce permissiveness for ONNV infection, thereby favoring elimination of infection and sterile immunity. Stochastic and genetic effects are not mutually exclusive explanations, because both could apply, depending on the penetrance of putative genetic factors. Host genetic influence on *Plasmodium* infection has been described, for example [31,32], but to our knowledge genetic association studies for virus infection in *Anopheles* have not been reported.

The silencing of *Rel2* alone allowed a significant elevated viral titer but did not alter infection prevalence. It is not clear why the silencing of *Rel2* alone did not alter ONNV infection prevalence, while the silencing of *Rel2* in the absence of Rin complemented the loss of Rin by reversing the silenced *Rin* phenotype. A possible technical explanation is that infection prevalence in the dsGFP-treated control mosquitoes was close to 80%, and thus the experimental design may have been better powered to detect decreased prevalence but lacked sufficient power to robustly detect a significant further prevalence increase above 80%. An alternative explanation could be that depletion of different antiviral pathways such as Imd or JAK/STAT can individually result in elevated viral titer, but that complete elimination and sterile immunity requires the integrated effect of multiple antiviral pathways. Further work using infections at different infectious viral titers would be required to distinguish between these possibilities. However, taken together, the results indicate that the proviral effect of Rin for ONNV infection is mediated at least in large part by a Rin-dependent inhibition of Imd pathway activity.

Similar to observations with Rin in *Aedes* and G3BPs in mammals [14], Rin of *Anopheles* is also able to colocalize and interact with nsP3 of ONNV, indicating the wide conservation of the Rin/G3BP interaction with alphavirus nsP3, although the mechanism is poorly understood in any system. Using proteomic methods, we identified a total of 63 protein partners in the Rin functional complex in *Anopheles* cells, most of them without known function, which highlights the complexity and novelty of the Rin network. Another recent study identified 193 *Anopheles* protein partners that can interact with nsP3 of ONNV and CHIKV [33]. That study used nsP3 as pulldown bait while in our study Rin was the bait, to focus on proteins in the Rin complex. There were no proteins identified in common in our dataset and Byers et al., either in presence or absence of nsP3, although the nsP3 bait in Byers et al. did pull down Rin. However, there are several differences between the studies. The main one is the difference in the baits, which, based on the results, may not behave in a commutative fashion with cellular proteins, that is, they may not attract the same partner complex when presented in either order. Also, the cellular environments were different, because we used the 4a-3A cell line, while Byers et al. used Sua4.0 cells. The 4a-3A cell line is fixed for the 2L+ allele of the 2La chromosome inversion, while Sua4.0 is fixed for the alternate 2La allele [34]. The 2La inversion locks approximately 10% of the genome in two highly diverged alternate allelic forms, which are genetically associated with significant difference in physiological susceptibility to *Plasmodium* infection [35]. The association of the 2La inversion genotype for virus infection phenotype has not been reported to our knowledge, but it would be interesting to do so. Thus, there are both biological and technical differences between the two studies that mean they are not necessarily comparable, and more information will be required to understand the different outcomes. A common theme in both studies, though, is the strong suggestion that nsP3 needs Rin to interact with host protein partners that nsP3 could not interact with by itself. Thus, ONNV nsP3 uses Rin as a molecular adaptor to target components of the host cellular machinery.

Our proteomic data suggest that Rin binding partners may be involved in processes of RNA metabolism and protein synthesis. Interestingly, our proteomic results do not find Rin interacting directly with known proteins in the immune pathways that we show it can modulate, except for Ago2 in the RNAi pathway. The observation that Rin does not directly bind known immune pathway proteins appears consistent with a report that Drosophila Rin differentially stabilizes certain mRNAs and thereby promotes translation of particular classes of genes, probably by binding and stabilizing particular classes of polysomes [17]. Thus, on this interpretation, the immune genes in this study for which transcript abundance is modified by Rin activity may not be regulated at the level of transcription rate, but rather Rin may act post-transcriptionally by preferentially enhancing the mRNA stability of specific mRNA classes, thereby increasing the steady-state transcript abundance of the mRNAs. In our results, the presence of ONNV nsP3 modifies the partner profile of the Rin complex, while still allowing Rin to interact with most of its protein partners. These results taken together suggest that the interaction of viral nsP3 with Rin, by altering the Rin-associated protein complex, changes the Rin biological effect upon at least the immune genes that we showed are influenced by Rin.

The mechanism of action of the Rin-associated protein partners, or the effect of nsP3, is not known. Indeed, half of the Rin-associated proteins in either condition do not have known or predicted functions, complicating interpretation or even speculation. The highest priority for further study would be the eight Rin-associated proteins whose presence is conditional upon the presence of nsP3 (of which five have unknown function). For this study, it would first be necessary to determine concordance of transcript abundance and protein levels, which would require antibody production for the target proteins. The antibodies and other reagents could be used in cell biological experiments to try to unravel the unknown functions of the proteins. Moreover, the functional interactions between partners or subunits in a protein complex can be complex, with potential functional redundancy or compensation among partners, such that detection of an infection phenotype by silencing individual partners may be reduced or masked, thus requiring combinatorial silencing of multiple partners and infection experiments. Alternately, in the absence of any compensation for partner loss, the silencing of any partner would then generate a dominant loss-of-function of the entire protein complex, which would also not be very informative in itself. Thus, functional analysis of the roles of candidate protein partners in a functional complex, particularly when most partners lack functional annotation, would represent a challenging study, and falls beyond the current scope.

The proviral role of G3BPs on alphavirus infection has primarily been studied during the RNA replication steps of the viral genome, particularly in mammalian host cells [19,25,36]. G3BPs in mammals have been shown to influence the switch from viral genome replication to genome translation [19]. However, the role of G3BPs in immunity during a viral infection is poorly understood, and prior to the current study has, to our knowledge, not previously been examined in the mosquito vectors. This study reveals a novel link between Rin, immunity and viral infection and could be extrapolated to Rin in other mosquito species and to G3BPs in mammals. The potential relevance of the current Rin results for G3BPs in other organisms is particularly strengthened by previous observations in vertebrate cells that G3BPs may influence components of the NF-kappa B pathway [20Rin], although the significance for host immunity was not examined. Here, we show that mosquito Imd, which is controlled by the NF-kappa B protein Rel2, is strongly controlled by the mosquito G3BP protein, and we demonstrate that the alphavirus ONNV targets this attribute of Rin in order to undermine host immune function. This mechanism underlies the proviral function of Rin in the mosquito, and because G3BPs in other studied systems are proviral for other alphaviruses, it seems likely that a similar mechanism of G3BP-mediated viral immune subversion could operate widely. Further work in other host-alphavirus systems will be necessary to uncover the similarities and differences with our results.

The multiplicity of G3BP/Rin function in cell metabolism make them a target of choice for viral manipulation. However, because of the multiplicity of Rin cellular functions, it would be a difficult target for therapeutic treatment in humans due to the risk of off-target secondary effects. However, dissecting the many partners of Rin with yet-unknown function could point to promising new therapeutic targets.

Finally, we observe that the study of arbovirus-host interactions in *Anopheles* has been relatively neglected in favor of the extensive research on *Anopheles* interactions with malaria parasites, and it is not understood why *Anopheles* mosquitoes in nature apparently do not serve as important vectors for arbovirus transmission as compared to *Aedes* and *Culex*. Behavior and ecology are probably not an explanation, because *Anopheles* vectors of human malaria display high human host preference, are sympatric with *Aedes* vectors of dengue and other viruses, and bite the same arbovirus-infected people as *Aedes*. We hypothesize that at least part of the explanation could lie in the efficiency of antiviral immunity, such that arbovirus infection of *Aedes* may pass relatively unobstructed, while only ONNV has decoded a pathway allowing transmission through *Anopheles*. Nevertheless, *Anopheles* mosquitoes harbor a complex natural virome of RNA viruses [37] and a number of pathogenic arboviruses have been isolated from *Anopheles* mosquitoes [7], for example, Rift Valley Fever virus (RVFV, genus Phlebovirus, family *Bunyavirales*) [38] and Japanese encephalitis virus (JEV, genus Flavivirus, family *Flaviviridae*) [39]. These observations highlight the importance of studying *Anopheles*-virus interactions. With changing climatic and ecological conditions, movement of invasive *Anopheles* into new environments [40], and exposure of vector populations to new potential pathogens, it is relevant to ask about the risk that arboviruses other than ONNV could adapt to *Anopheles* mosquitoes as transmission vectors.

## Materials and methods

### Ethics statement

The protocol for the ethical treatment of the animals used in this study was approved by the research animal ethics committee of the Institut Pasteur, “C2EA-89 CETEA Institut Pasteur” as protocol number 202195.02. The Institut Pasteur ethics committee is authorized by the French Ministry of Higher Education and Research (MESR) under French law N° 2001–486, which is aligned with Directive 2010/63/EU of the European Commission on the protection of animals used for scientific purposes. The study was performed using practices and conditions approved by the Institut Pasteur Biosafety Committee as protocol number CHSCT 14.114.

### Mosquito, cell and virus strains

The *Anopheles coluzzii* Ngousso colony was initiated in Cameroon in 2006 and has been maintained at the Institut Pasteur Center for the Production and Infection of *Anopheles* (CEPIA) facility since 2008. Mosquitoes are maintained at 26°C, 70% humidity. Female mosquitoes used for all experiments are less than 5 days post-emergence.

*Anopheles coluzzii* 4a3A cells originated from larvae and spontaneously immortalized [41]. 4a3A cells are maintained at 27°C without CO_2_ in Insect-XPRESS Protein-free Insect Cell Medium complemented with heat-inactivated Fetal Calf Serum (FCS) (ThermoFisher Scientific). The C6/36 cell line (ATCC) [42] was used for viral titration and is maintained in Leibovitz’s L-15 medium (ThermoFisher Scientific) supplemented with MEM non-essential amino acid (ThermoFisher Scientific) and complemented with heat-inactivated Fetal Calf Serum (ThermoFisher Scientific) at 28°C without CO_2_. Baby hamster kidney cell line (BHK-21) was used to produce virus particles from infectious clones and are maintained in Dulbecco’s Modified Eagle Medium (DMEM) (1X) + GlutaMax media (ThermoFisher Scientific) complemented with heat-inactivated Fetal Calf Serum (ThermoFisher Scientific) at 37°C with 5% CO_2_.

The infectious clone of ONNV originated from ONNV strain Igbo Ora-IBH10964 (Igbo-Ora strain) and was isolated from a human febrile patient during the epidemic of 1966 in Nigeria (accession number AF079457) [43]. ONNV was either produced on *Anopheles* 4a3A cells and viral titer done on kidney epithelial cells from African green monkey (Vero cells) were measured at 7.3 x 10^5^ plaque forming units/mL (pfu/mL) for cell infection. Mosquito infection used infectious ONNV produced on BHK-21 cells and the titer measured on C6/36 cells is 5.64 x 10^7^ FFU/mL. Infectious clone cDNA was linearized by PmeI, and viral RNA was transcribed using T7 RNA polymerase (Ambion mMESSAGE mMACHINE SP6 Kit). Transcribed RNA was electroporated into BHK-21 or 4a3A cells. Virus was recovered after 72 h and titer on C6/36 cells.

### Primers and plasmids

The dsRNA amplicons were constructed using primers (given in S1 Table) derived from the *An. gambiae* PEST reference genome available in the VectorBase database. DsRNAs targeting GFP act as a control for the dsRNA treatment. DsRNAs were synthesized using T7 primers and the transcription kit of MEGAscript T7 (Invitrogen) following manufacturer instructions.

Plasmids used for colocalization, and streptavidin pull-down assay were constructed using *Rin* codon-optimized AGAP000403 sequences and ONNV nsP3 protein from IBH10964 strains graciously provided by Andres Merits, University of Tartu, Estonia. A C-terminal streptavidin tag II (W-S-H-P-Q-F-E-K) was added at the end of *Rin* sequences. For their expression in 4a3A cells, these genes are expressed under the Actin promoter (Ac5) in the plasmid pAc5.1. 4a3a cells lines were transfected using lipofectamine LTX (ThermoFisher Scientific) with 0.25 µg to 0.5 of plasmids depending on the number of cells used.

### RNA extraction, cDNA synthesis and qPCR analysis

RNA was extracted from 4a3A cells or mosquitoes using Trizol reagent (Sigma Aldrich) and RNA miniprep kit (Ozyme). Complementary DNAs were synthesized with M-MLV transcriptase inverse kit (Invitrogen) using 1 µg of RNA. Quantitative polymerase chain reaction (qPCR) primers used for detection of the gene silencing and for analyzing the transcript level of immune genes and viral RNA are shown in S1 Table. The qPCR primers were checked for specificity prior to the analysis. All qPCRs were performed using SYBR green supermix (KAPA SYBR FAST ABI, Sigma-Aldrich) and the CFX96 Touch Real-Time PCR Detection System (BioRad). The ribosomal protein rpS7 gene was used as the internal control and the analysis of transcript relative expression to rpS7 was performed according to the 2 − ΔΔCt method. PCR cycle conditions were 95°C for 10 min, then 39 cycles of 95°C for 15 sec, 60°C for 1 min.

### Antibodies and fluorescent labels

Sera of rabbit containing polyclonal rabbit antibodies provided by Andres Merits, University of Tartu, targeting the macro and the AUD domain of nsP3 of ONNV were used at 1:100 overnight at +4°C for colocalization and western blot assay. Secondary goat anti-Rabbit IgG (H + L) antibodies (Invitrogen) conjugated with Alexa Fluor 555 were used during colocalization assay at 1:500 for 45 min at room temperature (RT) in 0.2% Triton X-100, 1% BSA, and PBS 1X. Secondary antibodies anti-rabbit conjugated with HRP (Invitrogen) were used for Western blot at 1:8000 for 1 h at RT in 5% BSA in PBS 1X. Monoclonal antibodies targeting the streptavidin tag II and conjugated with HRP (IBA Lifesciences) were used at 1:10000 for 1 h at RT during Western blot in 5% BSA in PBS 1X. Streptavidin Strep-Tactin molecule conjugated with dye-649 (IBA Lifesciences) were used at 1:100 for 3 h at RT during colocalization assay in 0.2% Triton X-100, 1% BSA, and PBS 1X. DAPI (4', 6-diamidino-2 '-phenylindole, dihydrochloride) (ThermoScientific) was used at 1:1000 for 10 min at RT in PBS 1X for staining the nucleus of 4a3A cells during nsP3-Rin cellular colocalization assays. Detection of viral titration used monoclonal mouse antibodies against CHIKV Virus-like particles (The Native Antigen Company), diluted at 1:1000 in 0.1% BSA PBS 1X solution for 45 min at 37°C. Secondary goat antibodies against mouse IgG and IgM (H + L) conjugated with Alexa 488 (Invitrogen) were diluted at 1:500 in PBS 1X and incubated at 37°C for 30 min.

### Gene silencing

For gene silencing in mosquitoes, 500 ng of dsRNA in a final volume of 70 nL was injected into the thorax of cold-anesthetized 1–2 d old *An. coluzzii* females using a Nanoject II injector (Drummond Scientific) and glass capillary needle as previously described [28]. The gene silencing efficiency was verified by RT-qPCR using pooled RNA from six unfed mosquitoes collected 2 days after dsRNA injection, and is presented for all figures in S1 File. For gene silencing genes in cultured cells, 1 × 10e5 *An. coluzzii* 4A3A cells were seeded and left to adhere overnight. Cells were incubated for 30 min on a rocker with 5000 ng of dsRNA in 200 µL of Insect Xpress media without FBS. After incubation, 300 µL of Insect Xpress media with 10% (vol/vol) FBS was added, and cells were incubated until collection or infection.

### Cell infection

The 4a3A cell line was infected with ONNV at a standard multiplicity of infection (MOI) of 0.01 (except as noted for MOI 0.5 for nsP3-Rin cellular colocalization experiments) for 1 h at 28°C without CO_2_ in Insect-X-Press media without FCS. After 1 hour of infection, cells were washed three times with Insect-X-Press without FCS. Then 2% FCS Insect-X-Press media were added to cells for the time of incubation. Cells were then collected at 24h or 48h post-infection.

### Infectious blood feeding

The experimental protocol is based on published methods [44]. The infectious blood meal was 2/3 vol/vol rabbit erythrocytes and 1/3 vol/vol of viral solution for a final titer of 10^7^ FFU/mL. Adenosine triphosphate (ATP) at 10 mM was also added to the infectious blood. Infectious blood was fed to mosquitoes using the Hemotek system with artificial skin maintained at 37°C. Mosquitoes were fed for 1 h, and unfed females were removed. Fed mosquitoes were maintained at 28°C and 80–90% humidity with access to 10% sugar solution. At three days post-infectious feeding, abdomens and thoraces of female mosquitoes were separated, homogenized, and centrifuged at +4°C for 5 min at 10,000 rpm. Supernatants were collected and stored at -80°C until analysis.

### Viral titration by focus formation assay

Only infected mosquitoes were analyzed for the determination of viral titer, to avoid conflating measures of infection prevalence and intensity. *Ae. albopictus* C6/36 cells were seeded in 96-well plates at 1.25 x 10^6^ cells/ Leibovitz’s L-15 medium (ThermoFisher Scientific) supplemented with MEM non-essential amino acids (ThermoFisher Scientific) with 10% of FBS and incubated at 28°C without CO_2_, 48h prior to titration. Virus-containing samples were serially diluted, and 50µL of the dilutions were added to cells in one well. After one hour of incubation at 28°C, 150 µL of 1:1 4% carboxymethylcellulose (CMC) and L-15 containing 5% FBS was layered onto cells, along with antibiotic-antifungal solution (Life Technologies) at a final concentration of 1.5X. Plates were incubated at 28°C without CO_2_ for 3 days, then cells were fixed using 3.4% formaldehyde in PBS 1X for 20 min at room temperature. Cells were then washed three times in PBS 1X and stained with primary antibodies. After three washes with PBS 1X, cells were stained with secondary antibodies. Viral titers were counted by observing foci using a fluorescent microscope and were expressed as focus forming units/mL (FFU/mL).

### Confocal microscopy

Cells were seeded in µ-slide 8-well plate (Ibidi GmbH) and transfected using lipofectamine with 0.25 µg of plasmids. Two days post-transfection, cells were fixated with 4% formaldehyde for 20 min at RT. Cells were then blocked and permeabilized using a solution of 0.2% Triton X-100, 1% BSA, PBS 1X for 1 h at RT, and were stained with antibodies diluted in 0.2% of Triton X-100, 1% BSA, and PBS 1X. Cells were washed using 0.2% Triton X-100 1% BSA PBS 1X. Colocalization analysis in 4a3A used a laser-scanning confocal microscope (LSM700, Carl Zeiss Jena) at the UTechS Photonic BioImaging C2RT platform (Institut Pasteur). Image acquisition parameters were as follows: 63X oil-objective, frame size 512x512, pixel dwell 1.57 µsec, scan time 5.78 sec, 16-bit depth, image size 32.9x32.9 µm and 0.06 µm pixel size. Laser parameters were as follows: the DAPI laser (450 nm) had an intensity of 2, a pinhole diameter of 0.6 µm, a master gain of 730 and a digital gain of 1. The Red A555 laser (514 nm) had an intensity of 2.6, a pinhole diameter of 0.6 µm, a master gain of 711 and a digital gain of 1. The Far-Red D647 laser (633nm) had an intensity of 2, a pinhole diameter of 0.8 µm, a master gain of 700 and a digital gain of 1. Images were analyzed using Fiji software [45]. Background signals were calculated with the mean of signals from 5 pictures of the negative controls of each fluorophore. Mean of background signals were then removed from the analyzed pictures. Colocalization was quantified by counting the number of cells with colocalization pattern as compared to cells co-transfected (harboring both signals). Between 30 and 50 cells were counted in the analysis.

### Streptavidin-pull down assay

First, 0.5 µg of plasmid constructs were transfected in 4a3A cells using lipofectamine LTX with Plus Reagent (ThermoFisher Scientific). Two days post-transfection, cells lysis was performed using cell lysis buffer (containing Tris 1 M, NaCl 5M, EDTA, Igepal, Protease inhibitor and Phosphatase inhibitor) for 30 min at +4°C and samples were centrifuged at 16,000g for 15 min at +4°C. Pull-down assay was performed using MagStrep type 3 beads (Strep-Tactin XT coated magnetic beads, 5% (v/v) suspension, IBA Lifesciences) with a binding capacity up to 0.85 nmol/μL beads (corresponding to 25.5 μg of a 30 kDa protein). Beads were first washed with cell lysis buffer then cell lysate was added on the beads and incubated for 2 h at +4°C on a tube rotator. After 2 h of incubation, lysates were removed, and washes were performed using 1X W buffer (IBA Lifesciences). Following washes, either on-beads digestion at the Proteomic platform is done for Mass spectrometry analysis or the elution of proteins is performed using 1X BXT buffer containing biotin (IBA Lifesciences) for 10 min. Eluates are then analyzed by Western Blot in order to detect strep-tag proteins (Rin or GFP, bait) and partners (nsP3, prey) using specific antibodies.

Protein samples were reduced in 1X DTT and heated at 95°C for 5 min. Then samples were mixed with XT sample buffer (Bio-Rad) and loaded on 4–12% Criterion SDS-PAGE gels (Bio-Rad). SDS-PAGE is run at 100 V for 10 min (stacking gel) and 150 V for 1 h (running gel) in NOVEX NUPAGE MOPS SDS Running Buffer 20X (Life Technologies). Following electrophoresis, proteins contained on gel are transferred onto a 0.2 µm nitrocellulose membrane (Bio-Rad) using Bio-Rad Trans-Blot Turbo Transfer System. Transfer program is run for 7 min at 2.5A and 25V. After transfer, immunoblots are blocked in a solution containing 5% of Bovine Serum Albumin (BSA) in TBS 1X (Tris-buffered saline, 0.1% Tween 20). Then, immunoblots were probed with antibodies at specific dilutions in 5% BSA TBS 1X. Detection step was performed using the Enhanced chemiluminescence (ECL) system (Clarity Western ECL substrate, BioRad) following manufacturer instruction. Blots were photographed and revealed taking one picture every second for 10 sec using ChemiDoc Imaging System (Bio-Rad). Blots were then annotated, and molecular weights were measured related to the protein molecular weight marker (Precision PlusProtein All Blue Standards, BioRad) using the Image Lab analysis software (BioRad). The molecular weight marker lane is labeled “M” on relevant blots. A positive control was performed using a commercial GFP-strep (Green Fluorescent Protein) (IBA LifeSciences) in order to confirm the success of the Western Blot.

### Mass spectrometry analysis

The products of the streptavidin-pull down assay were analyzed for differential protein abundance by liquid chromatography mass spectrometry (LC-MS/MS). First, for on-bead digestion of samples, proteins were resuspended in 50 mM ammonium bicarbonate pH 8.0 and reduced for 30 min at room temperature (RT) with 5 mM TCEP and then alkylated at 50 mM iodoacetamide (Sigma) for 30 min at RT in the dark. Proteins were then digested with 0.5 µg Sequencing Grade Modified Trypsin (Promega). Following overnight digestion at 37°C with agitation at 800 rpm in a Thermomixer C (Eppendorf), the sample tubes were placed on a magnet for 1 min, and the supernatant containing the released peptides was transferred to a new tube. Resulting peptides were acidified at 1% with formic acid, desalted with an AssayMAP Bravo robot using C18 column and AssayMAP Peptide Cleanup Protocol (Agilent). All samples were dried in a Speed-Vac and peptides were resuspended in 2% ACN, 0.1% FA prior to LC-MS/MS analysis. LC-MS/MS analysis of digested peptides was performed on an Orbitrap Eclipse mass spectrometer (Thermo Fisher Scientific) coupled to an EASY-nLC 1000 (Thermo Fisher Scientific). Peptides were loaded (at constant pressure of 900 bars) and separated at 250 nl.min-1 on a home-made C18, 30 cm capillary column picotip silica emitter tip (75 μm diameter filled with 1.9 μm Reprosil-Pur Basic C18-HD resin, (Dr. Maisch HPLC GmbH) equilibrated in solvent A (2% ACN, 0.1% FA). Peptides were eluted using a gradient of solvent B (80% ACN, 0.1% FA) from 2% to 7% in 3 min, 7% to 31% in 42 min, 31% to 62% in 10 min, 62% to 95% in 5 min (total length of the chromatographic run was 70 min). The column was equilibrated with 10 µL of A at 900 bars. Mass spectra were acquired in data-dependent acquisition mode with the XCalibur software (Thermo Fisher Scientific). MS spectra were acquired at orbitrap resolution of 60k (at m/z 400), AGC target 800000, a custom max injection time. The scan range was limited from 300 to 1500 m/z. Peptide fragmentation was performed using higher-energy collision dissociation (HCD) with the energy set at 27 NCE. The MS/MS spectra acquired at a resolution of 30k (at m/z 400). Isolation window was set at 1.6 m/z. All acquisitions were done in profile and positive mode. Dynamic exclusion was employed within 30 sec. Raw data and statistical analyses of mass spectrometry analysis are presented in S2 File.

Peptide identification and statistical analysis were as follows. Raw data were analyzed using MaxQuant software version 2.0.3.0 [46] using the Andromeda search engine [47]. The MS/MS spectra were searched against a custom database from the Vectorbase repository for the PEST reference genome of *Anopheles gambiae* (download 25/04/2022). Usual known mass spectrometry contaminants and reversed sequences of all entries were included in the search. The Andromeda search was performed choosing trypsin as specific enzyme with a maximum number of two missed cleavages. Possible modifications included carbamidomethylation (Cys, fixed), oxidation (Met, variable), Nter acetylation (variable). The mass tolerance in MS was set to 20 ppm for the first search then 4.5 ppm for the main search and 20 ppm for the MS/MS. Maximum peptide charge was set to seven and seven amino acids were required as minimum peptide length. The “match between runs” feature was applied for samples having the same experimental condition with a maximal retention time window of 0.7 minute. One unique peptide to the protein group was required for the protein identification. A false discovery rate (FDR) cutoff of 1% was applied at the peptide and protein levels. For the differential analyses of one condition versus another, Intensity-Based Absolute Quantification (IBAQ) quantification was used. Proteins identified in the reverse and contaminant databases and proteins “only identified by site” were first discarded from the list of identified proteins. Only proteins with at least three quantified intensities in a condition were kept. The proteins of interest are therefore those which emerge from this statistical analysis supplemented by those which are considered to be present from one condition and absent in another.

### Statistical analysis

All experiments are the results of at least three independent replicate experiments. For RT-qPCR analysis of RNA transcript levels relative to rpS7, the 2 − ΔΔCt method was used, and the difference in deltaCt distribution across biological replicates was statistically tested using unpaired t-test. Differences in the infection rates were analyzed using a chi-squared test. A two-tailed non-parametric unpaired Mann-Whitney test was performed to assess the statistical significance of the difference in viral titer in mosquitoes. The P-values were assessed with a null distribution. The P-values were considered significant if p < 0.05 (*p < 0.05; **p < 0.01; ***p < 0.005, ****p < 0.0001). Bar plots were created using GraphPad Prism (Dotmatics Software).

## Supporting information

S1 Fig Rin is a proviral factor for ONNV infection in *Anopheles* 4a3A cells. The effect of Rin silencing upon **(A**) viral RNA quantity and (**B**) infectious particle production are shown. Bars represent the level of transcript abundance of ONNV RNA **(A)** or focus-forming units **(B)** relative to dsGFP-treated controls (defined as 1.0), error bars indicate the SEM. Results are from three independent replicates. A two-tailed non-parametric unpaired Mann-Whitney test was performed to assess the statistical significance of the difference in viral titer in mosquitoes. Student t-test analysis, which compares the mean of two independent groups (control and treatment) were used to assess the statistical significance of the difference in transcript abundance. The P-values were considered significant if ** P < 0.01, *** P < 0.001, **** P < 0.0001, ns non-significant.
(PDF)

S2 Fig Rin transcript silencing efficiency by dsRin treatment of 4a3A cells. *Rin* transcript levels after dsRin treatment in naïve cells **(A)** or ONNV-infected cells **(B)** are shown at 24h (black bar) and 48h (grey bar) post-dsRNA exposure. Bars represent the level of transcript abundance of *Rin* relative to dsGFP-treated control (defined as 1.0), error bars indicate the SEM. Results are from three independent replicates. Student t-test analysis, which compares the mean of two independent groups (control and treatment) were used to assess the statistical significance of the difference in transcript abundance. The P-values were considered significant if ** P < 0.01, *** P < 0.001, **** P < 0.0001, ns non-significant.
(PDF)

S3 Fig Raw blot data for mass spectrometry replicates in 4a3A cells. **(A-B)** A blot was made from the eluates used for mass spectrometry and were stained with streptavidin-specific monoclonal antibodies **(A)** and nsP3-specific polyclonal antibodies **(B)**. 4a3A cells were transfected with 250 ng of plasmids encoding Rin-strep and/or nsP3 of ONNV. Two days post-transfection, cells were collected and a streptavidin-pull down assays for mass spectrometry using streptavidin Strep-Tactin beads were done on the cell lysate. SDS-PAGE was run on the eluate of the streptavidin-pull down then a Western blot was done to reveal the presence of Rin-strep and/or ONNV nsP3. Staining of the blot was done sequentially with streptavidin-specific antibodies **(A)** and then, without stripping the blot, with nsP3-specific antibodies **(B)**. The staining results of the elution of non-transfected control cells (NT), transfected with Rin-strep (Rin-strep, 89 kDa), nsP3 of ONNV (nsP3, 70 kDa) or co-transfected (Rin-strep + nsP3) are shown. A control corresponding to GFP-strep commercial protein (+, 25 kDa) is shown. The molecular weight marker (M) is shown, marker bands labeled in kiloDaltons (kDa). Samples on the blots are from four independent replicates (indicated as Rep 1, Rep 2, Rep 3 and Rep 4). A blot transfer artifact in the Rep 2 lanes distorted the Rin-strep band in (A), and rendered the nsP3 band present but very faint in (B).
(PDF)

S1 Table Primer Sequences. Primer Sequence for qPCR analysis, plasmid construction and dsRNA synthesis.
(DOCX)

S1 File Raw data for all figures and replicates. The infection results, gene silencing efficiency, and transcript fold-change raw data is presented for all individual replicates of all figures.
(XLSX)

S2 File Raw data and statistical analysis for mass spectrometry. Raw mass spectrometry data and statistical analyses underlying all results presented in Fig 6.
(ZIP)
