# Supplementary figures and images for: Rasputin/G3BP mediates subversion of antiviral immunity by o’nyong-nyong virus in Anopheles coluzzii
Source: PLoS Pathog. 2026 Jul 14;22(7):e1014423. doi: 10.1371/journal.ppat.1014423 (PMC13384397; doi:10.1371/journal.ppat.1014423)

# A. Viral RNA

# B. Infectious particles

## FIGURE S1

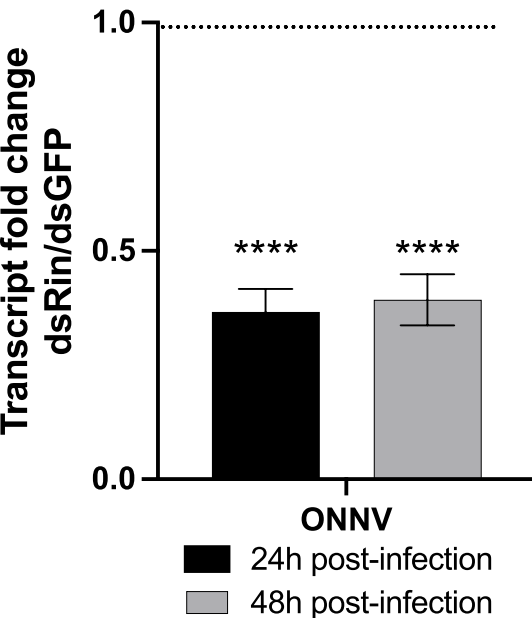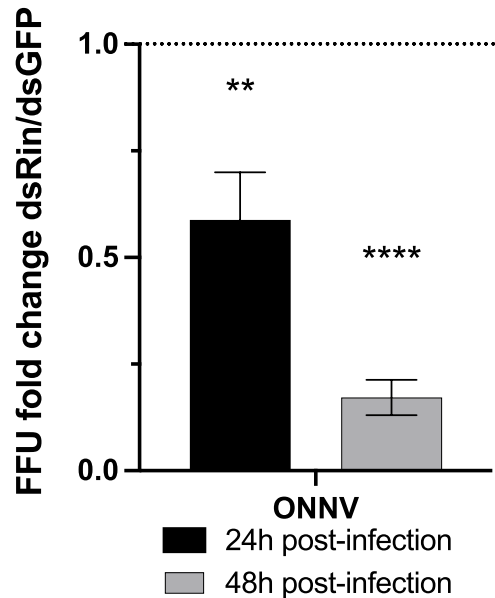

Supplement: S1 Fig — The effect of Rin silencing upon (A) viral RNA quantity and (B) infectious particle production are shown. Bars represent the level of transcript abundance of ONNV RNA (A) or focus-forming units (B) relative to dsGFP-treated controls (defined as 1.0), error bars indicate the SEM. Results are from three independent replicates. A two-tailed non-parametric unpaired Mann-Whitney test was performed to assess the statistical significance of the difference in viral titer in mosquitoes. Student t-test analysis, which compares the mean of two independent groups (control and treatment) were used to assess the statistical significance of the difference in transcript abundance. The P-values were considered significant if ** P < 0.01, *** P < 0.001, **** P < 0.0001, ns non-significant. (PDF) [file ppat.1014423.s001.pdf]

# A. Naive cells

# B. Infected cells

## FIGURE S2

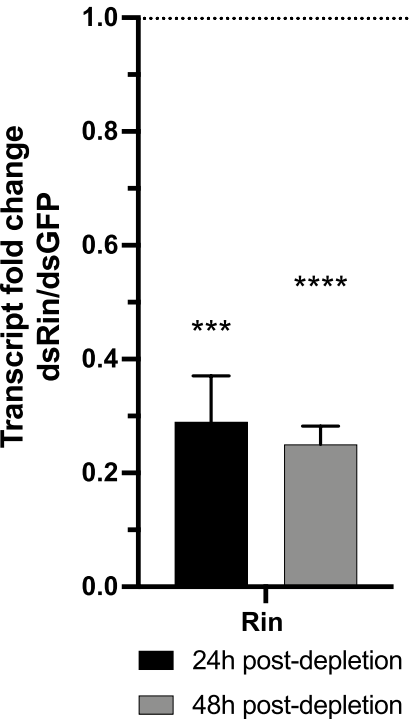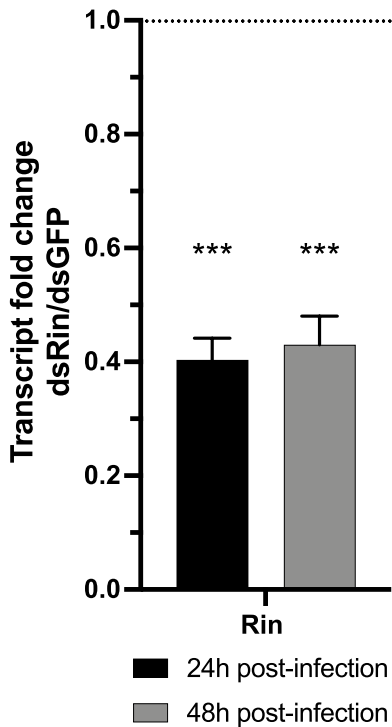

Supplement: S2 Fig — Rin transcript levels after dsRin treatment in naïve cells (A) or ONNV-infected cells (B) are shown at 24h (black bar) and 48h (grey bar) post-dsRNA exposure. Bars represent the level of transcript abundance of Rin relative to dsGFP-treated control (defined as 1.0), error bars indicate the SEM. Results are from three independent replicates. Student t-test analysis, which compares the mean of two independent groups (control and treatment) were used to assess the statistical significance of the difference in transcript abundance. The P-values were considered significant if ** P < 0.01, *** P < 0.001, **** P < 0.0001, ns non-significant. (PDF) [file ppat.1014423.s002.pdf]

**FIGURE S3**

**A.**

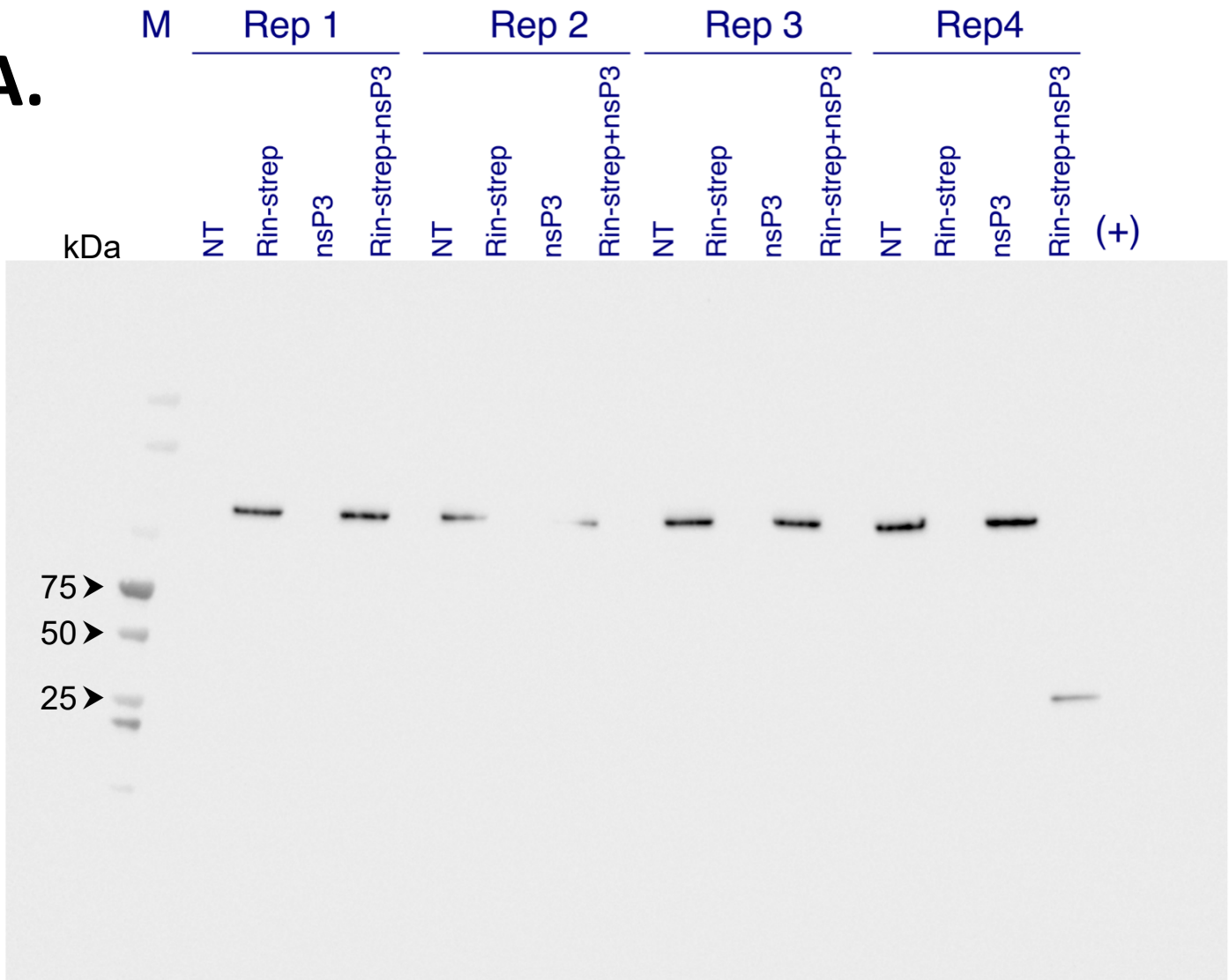

**B.**

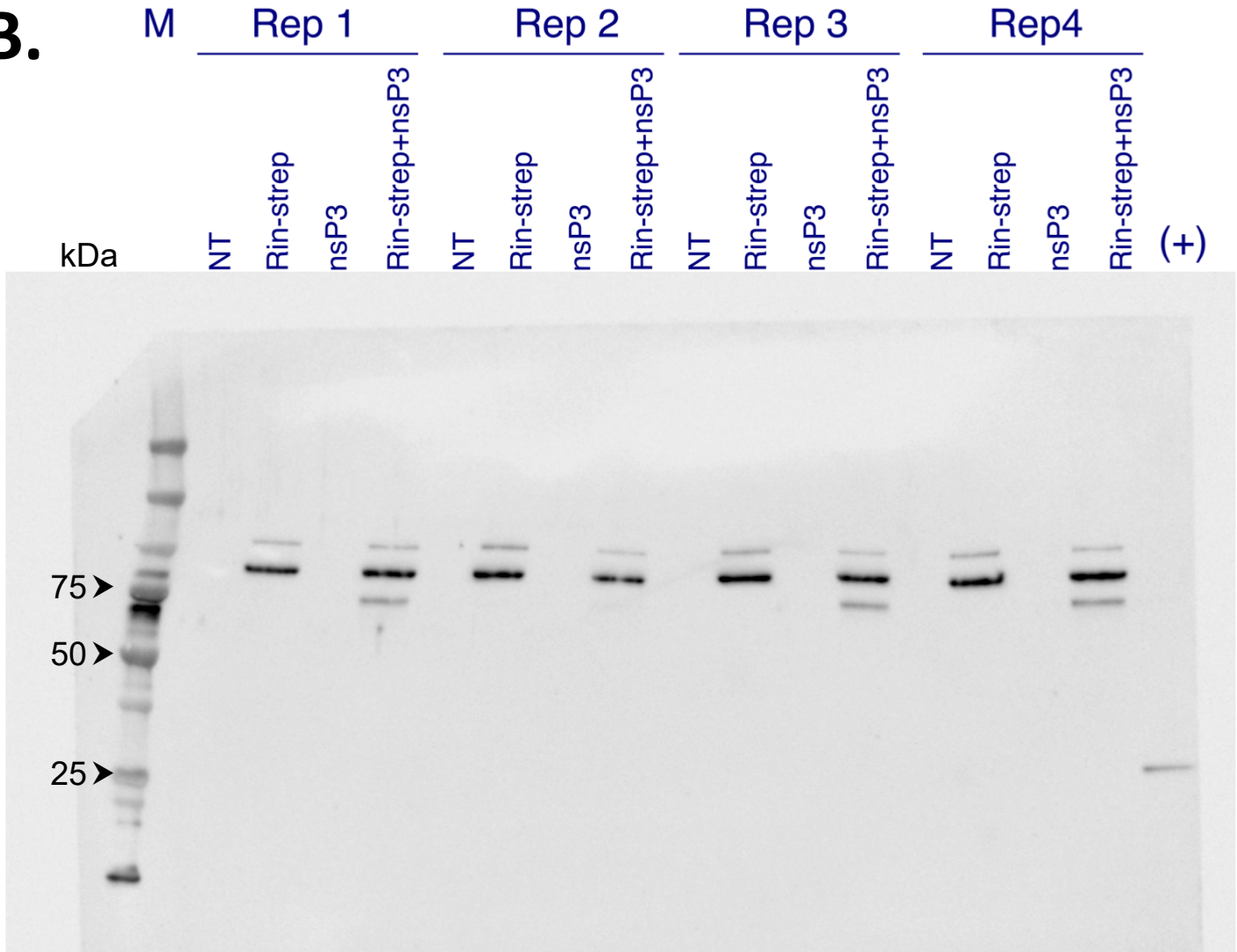

Supplement: S3 Fig — (A-B) A blot was made from the eluates used for mass spectrometry and were stained with streptavidin-specific monoclonal antibodies (A) and nsP3-specific polyclonal antibodies (B). 4a3A cells were transfected with 250 ng of plasmids encoding Rin-strep and/or nsP3 of ONNV. Two days post-transfection, cells were collected and a streptavidin-pull down assays for mass spectrometry using streptavidin Strep-Tactin beads were done on the cell lysate. SDS-PAGE was run on the eluate of the streptavidin-pull down then a Western blot was done to reveal the presence of Rin-strep and/or ONNV nsP3. Staining of the blot was done sequentially with streptavidin-specific antibodies (A) and then, without stripping the blot, with nsP3-specific antibodies (B). The staining results of the elution of non-transfected control cells (NT), transfected with Rin-strep (Rin-strep, 89 kDa), nsP3 of ONNV (nsP3, 70 kDa) or co-transfected (Rin-strep + nsP3) are shown. A control corresponding to GFP-strep commercial protein (+, 25 kDa) is shown. The molecular weight marker (M) is shown, marker bands labeled in kiloDaltons (kDa). Samples on the blots are from four independent replicates (indicated as Rep 1, Rep 2, Rep 3 and Rep 4). A blot transfer artifact in the Rep 2 lanes distorted the Rin-strep band in (A), and rendered the nsP3 band present but very faint in (B). (PDF) [file ppat.1014423.s003.pdf]
