# Supplementary material for: Rasputin/G3BP mediates subversion of antiviral immunity by o’nyong-nyong virus in Anopheles coluzzii
Source: PLoS Pathog. 2026 Jul 14;22(7):e1014423. doi: 10.1371/journal.ppat.1014423 (PMC13384397; doi:10.1371/journal.ppat.1014423)
Supplement: S1 Table — Primer Sequence for qPCR analysis, plasmid construction and dsRNA synthesis. (DOCX) [file ppat.1014423.s004.docx]

**Table S1. Primer Sequences**. Primer Sequence for qPCR analysis, plasmid construction and dsRNA synthesis.

**Primer Sequences for qPCR analysis**

| Target gene | Sequence primer Forward | Sequence primer Reverse |
| --- | --- | --- |
| rpS7 | ATGGTGGTCTGCTGGTTCTT | CACCGCCGTGTACGATGCCA |
| rpS17 | TCGAAGAAGTGGCCATCATTC | AGGAACGTGAGCGCAGAGA |
| Rin | TCGGCCAGAAGCAGATACAG | AGATGTCGTTGTGCACGTAGT |
| P70 | GCTCTCCTCCGCACAAATCA | AAACGTGCAGTACGGGGTC |
| ONNV | ACTCCAGGAGAGTGCATCCA | CCTGCAACCCTCTTTCAGTC |
| CHIKV | ACTCCAGGAGAGTGCATCCA | CTACAGCCTCTCTTTAGTCTCTGG |
| APL1A | GACTGCAAGCCGAGATCGATACC | CATCCATCTGGTCCTTGAGCTTA |
| APL1C | AAGCAGGCTGAGTTGAGACAGG | ATGGTGGTCTGCTGGTTCTT |
| TEP3 | ACCGCCAGGCGTACGTGATGG | CAAACCTTTCTCAGTCGAGGT |
| TEP4 | GGACCTCCATAATGCGGTGGC | CGGGGAGATCTTTTCCGCCAG |
| Rel1 | ACCACAATGCAAGTGCTTTTC | CTGGTACTGGTTGGAGGGATTG |
| Rel2-F | ATTGCGGTCGACAATGACGC | TGCCCGGTGTGGCTCTTCGC |
| Rel2 | ACCGATACGGAAAGTGTGCT | CGGTGCTCCTCGTAATGACT |
| Cactus | CCAAGACAAGCGCATCTTG | AGAAGGAAAGAATCGTCAC |
| Rin Anopheles plasmid | TTACGGAGCTCCGCCATTG | CTGGTCCAGCTTGGATTTC |

**Primer Sequence for plasmid construction and dsRNA synthesis**

| Target gene | Sequence primer Forward | Sequence primer Reverse |
| --- | --- | --- |
| T7-GFP | TAATACGACTCACTATAGGGCATGGTGAGCAAGGGCGAG | TAATACGACTCACTATAGGGCCCTTGAAGAAGATGGTGCGC |
| T7-Rin | TAATACGACTCACTATAGGCTGGTCAAGTCGGGCGTTAG | TAATACGACTCACTATAGGAGAACGCTGCCGTTGGTGGA |
| T7-Rel2-F | TAATACGACTCACTATAGGGATCCGACGCAACGATA | TAATACGACTCACTATAGGGACCGCAATGTGAAGGA |
| T7-Rel2 | TAATACGACTCACTATAGGGCACAGGCACACCTGAT | TAATACGACTCACTATAGGGCAACAGCAGCAACAAC |
